# Management of Multiple Myeloma and Usage of Bortezomib: Perspective from India and Ukraine

**DOI:** 10.3389/fonc.2016.00243

**Published:** 2016-11-21

**Authors:** India and Ukraine Haemato-oncology Group, Dharma Choudhary, Amit Garg, Mykhaylo Morgunskyy, Yogesh Belagali, Namita Gupta, Shyam Prasad Akku

**Affiliations:** ^1^Global Medical Affairs, Dr. Reddy’s Laboratories Ltd., Hyderabad, India; ^2^Global Medical Affairs, Dr. Reddy’s Laboratories Ltd., Kyiv, Ukraine

**Keywords:** multiple myeloma, bortezomib, generics, India, Ukraine

## Abstract

Novel treatment strategies have remarkably improved the multiple myeloma (MM) patients’ survival, with associated increased costs. A joint panel meet of international experts from India and Ukraine was held in New Delhi on May 19, 2016 focusing on MM management, bortezomib role, unmet medical needs, and current challenges. The health-care system for oncology in India is majorly private vs. government-based in Ukraine. In India, electrophoresis, serum-free light chain assays, bone marrow tests, and X-rays are available modes of diagnosis. Despite of the numerous cancer centers and stem cell transplant centers, most patients do not prefer transplant owing to its high-cost and social stigma. Majority of the patients are treated with bortezomib or lenalidomide-based regimens. Most patients buy drug themselves. The expanding generic drugs market is a ray of hope for the affordable drugs. In Ukraine, immuno-fixation, bone marrow analysis, and magnetic resonance imaging are common diagnostic modalities. Due to high cost, only few patients undergo transplant. Bortezomib-based regimens are preferred in most of the patients; however, usage is limited due to high costs and lack of funds. Thalidomide-based regimens are used for maintenance therapy due to affordability. In case of relapsed MM, bortezomib is preferred in triple therapy; however, more affordable option is cyclophosphamide, thalidomide, and dexamethasone (CTD). Issues, such as cost containment, common treatment strategies, enhanced collaboration, and improved health-care access, need immediate attention. High-quality generics access will improve outcomes and support health-care cost containment. Pharmacoeconomic studies and head-to-head trials are warranted to determine the cost-effectiveness and benefit of novel therapies in MM.

## Introduction

Multiple myeloma (MM) is a heterogeneous disorder which accounts for 10% of hematological cancers (1). Incidence of MM was found to be 2.1 (per 100,000 of people) in Ukraine, which is much lesser than in the United States (4.3) and Europe (4.1) (2) and almost on par with incidence in India, i.e., 1.2–1.8 per 100,000 of people (3).

There has been a remarkable improvement in the survival of MM patients in the last decades. This clinical progress was attributed to the availability of novel and target specific approach to treatment – proteasome inhibitors and immunomodulators (iMiDs), such as thalidomide, lenalidomide, and bortezomib and autologous stem cell transplantation (ASCT) (2).

Multiple myeloma poses a significant health-care challenge from both cost and quality of care perspective. In the US, the cost of MM is attributed to almost 9–10% of total cancer care costs. The rapidly increasing trend in costs is due to the development of novel agents, new diagnostic and treatment modalities, aging, and expanding populations, and increasing health-care costs (4).

Bortezomib, a first in class protease inhibitor (PI) was approved in 2003 for the treatment of progressive MM in patients who had received at least two prior therapies. In combination with melphalan and prednisone, it is also indicated for the treatment of adult patients with previously untreated MM who are not eligible for high-dose chemotherapy with hematopoietic stem cell transplantation. Currently, it has been recommended for primary induction therapy and also for consolidation and salvage therapy post relapse (5).

Since its development, bortezomib has come a long way in treating MM patients. Its wider therapeutic spectrum ranging from frontline induction to maintenance therapy has been increasingly explored in studies with the goal of achieving deeper responses, longer progression free survival (PFS), and possibly overall survival (OS). Bortezomib, in combination with other drugs, can also be reused in relapse setting (6).

Novel drugs, such as carfilzomib, Ixazomib, and pomalidomide, have also been introduced lately, leading to the bright future of MM patients. A few more drugs with novel mechanisms of action are under testing phase in clinical trials.

These drugs are used either alone or as a part of combination regimen to demonstrate substantial activity in terms of higher complete response (CR) rates than previous standard regimens.

There are several aspects, which need to be considered in the management of MM. A common treatment algorithm to standardize treatment worldwide, cost reduction for emerging treatment strategies, uniform adaptation of treatment guidelines, country-wide variations in outcome of MM, need for better care, and improved communication among treatment providers are some of these aspects. The concept of clonal diversity among patients needs attention while deciding the MM therapies. Moreover, there is an unmet medical need for more treatment options in relapsed or refractory disease, extramedullary disease, and those with high-risk cytogenetic profiles (4).

To address such issues, there is a need for collaborative effort. In this regard, a joint panel meet of international experts was held in New Delhi on May 19, 2016. Various experts in hematology and oncology were invited from India and Ukraine to share experiences from their respective countries in the management of MM, with special emphasis on the role of bortezomib in MM.

This paper reflects the management in MM patients, ushering toward the role of bortezomib alone or in combination with other drugs. The paper also focusses on analysis from various trials and discussing their implications in clinical practice. It briefs about the challenges, unmet medical needs discussed in the experts meeting, and finally discusses the management strategies followed in Ukraine, the differences from practices in India, with the current challenges faced by the physicians and patients in these developing countries.

## Role of Bortezomib in Multiple Myeloma

The management of MM patients and the choice of therapy (category 1 or category 2A drugs) as per the National Comprehensive Cancer Network (NCCN) guidelines (7) (Table 1) was an important point of discussion in the meeting. There was also a brief discussion on the key trials conducted on bortezomib (Table 2).

**Table 1:** **National Comprehensive Cancer Network (NCCN) Guidelines (7)**.

	Preferred regimens	Other regimens
Primary therapy for transplant candidates (assess for response after 2 cycles)	Bortezomib/dexamethasone (category 1) Bortezomib/cyclophosphamide/dexamethasone Bortezomib/doxorubicin/dexamethasone (category 1) Bortezomib/lenalidomide/dexamethasone Bortezomib/thalidomide/dexamethasone (category 1) Lenalidomide/dexamethasone (category 1)	Carfilzomib/lenalidomide/dexamethasone Dexamethasone (category 2B) Ixazomib/lenalidomide/dexamethasone Liposomal doxorubicin/vincristine/dexamethasone (DVD) (category 2B) Thalidomide/dexamethasone (category 2B)
Primary therapy for non-transplant candidates (assess for response after 2 cycles)	Bortezomib/dexamethasone Bortezomib/cyclophosphamide/dexamethasone Bortezomib/lenalidomide/dexamethasone (category 1) Lenalidomide/low-dose dexamethasone (category 1) Melphalan/prednisone/bortezomib (MPB) (category 1) Melphalan/prednisone/lenalidomide (MPL) (category 1) Melphalan/prednisone/thalidomide (MPT) (category 1)	Dexamethasone (category 2B) Ixazomib/lenalidomide/dexamethasone Liposomal doxorubicin/vincristine/dexamethasone (DVD) (category 2B) Melphalan/prednisone (MP) Thalidomide/dexamethasone (category 2B) Vincristine/doxorubicin/dexamethasone (VAD) (category 2B)
Maintenance therapy	Bortezomib Lenalidomide (category 1) Thalidomide (category 1)	Bortezomib + prednisone (category 2B) Bortezomib + thalidomide (category 2B) Interferon (category 2B) Steroids (category 2B) Thalidomide + prednisone (category 2B)

**Table 2:** **Selected trials discussed in the meeting**.

Clinical studies	Treatment arms	Sample size (no. of patients)	Primary efficacy measures
**Bortezomib: key clinical trials in previously untreated MM patients**			
VISTA TRIAL (11) (Phase 3 study)	BTZ 1.3 mg/m^2^ + MP vs. MP alone	682	TTP: 24.0 months (BTZ + MP)
			TTP: 16.6 months (MP alone)
IFM 2005-01 (12) (Phase 3 study)	VD (BTZ 1.3 mg/m^2^ + DEX 40 mg) vs. VAD	482	Post induction CR/nCR: 14.8% (VD)
			Post induction CR/nCR: 6.4% (VAD)
GIMEMA Italian Myeloma Network (13) (Phase 3 study)	VTD (BTZ 1.3 mg/m^2^ + THAL + DEX 40 mg) vs. TD	480	Post induction CR/nCR: 31% (VTD)
			Post induction CR/nCR: 11% (TD)
HOVON-65/GMMG-HD4 (14) (Phase 3 study)	PAD (BTZ 1.3 mg/m^2^ + DOX + DEX 40 mg) vs. VAD	827	PFS: 35 months (PAD)
			PFS: 28 months (VAD)
**Bortezomib: key clinical trials in relapsed/refractory multiple myeloma (RRMM)**			
SUMMIT (Phase 2 study) (15)	BTZ 1.3 mg/m^2^	193	ORR: 35%
CREST (16) (Phase 2 study)	BTZ 1.3 mg/m^2^	54	ORR: 50% (1.3 mg/m^2^)
	BTZ 1.0 mg/m^2^		ORR: 33% (1.0 mg/m^2^)
APEX TRIAL (17) (Phase 3 study)	BTZ 1.3 mg/m^2^ vs. high-dose DEX 40 mg	669	TTP: 6.22 months (BTZ)
			TTP: 3.49 months (high-dose DEX)
Pegylated liposomal doxorubicin + bortezomib (Phase 3 study) (18)	PLD 30 mg/m^2^ + BTZ 1.3 mg/m^2^ vs. BTZ 1.3 mg/m^2^	646	TTP: 9.3 months (PLD + BTZ)
			TTP: 6.5 months (BTZ)

*VISTA, Velcade as initial standard therapy in multiple myeloma: assessment with melphalan and prednisone; BTZ, bortezomib; MP: melphalan, prednisolone; TTP, time to progression; DEX, dexamethasone; VAD, vincristine, adriamycin, dexamethasone; CR/nCR, complete response/near complete response; THAL, thalidomide; DOX, doxorubicin; SUMMIT, study of uncontrolled multiple myeloma managed with proteasome inhibition therapy; CREST, clinical response and efficacy study of bortezomib in the treatment of relapsing multiple myeloma; APEX, assessment of proteasome inhibition for extending remissions; ORR, overall response rate; PLD, pegylated liposomal doxorubicin*.

**Figure 1:**
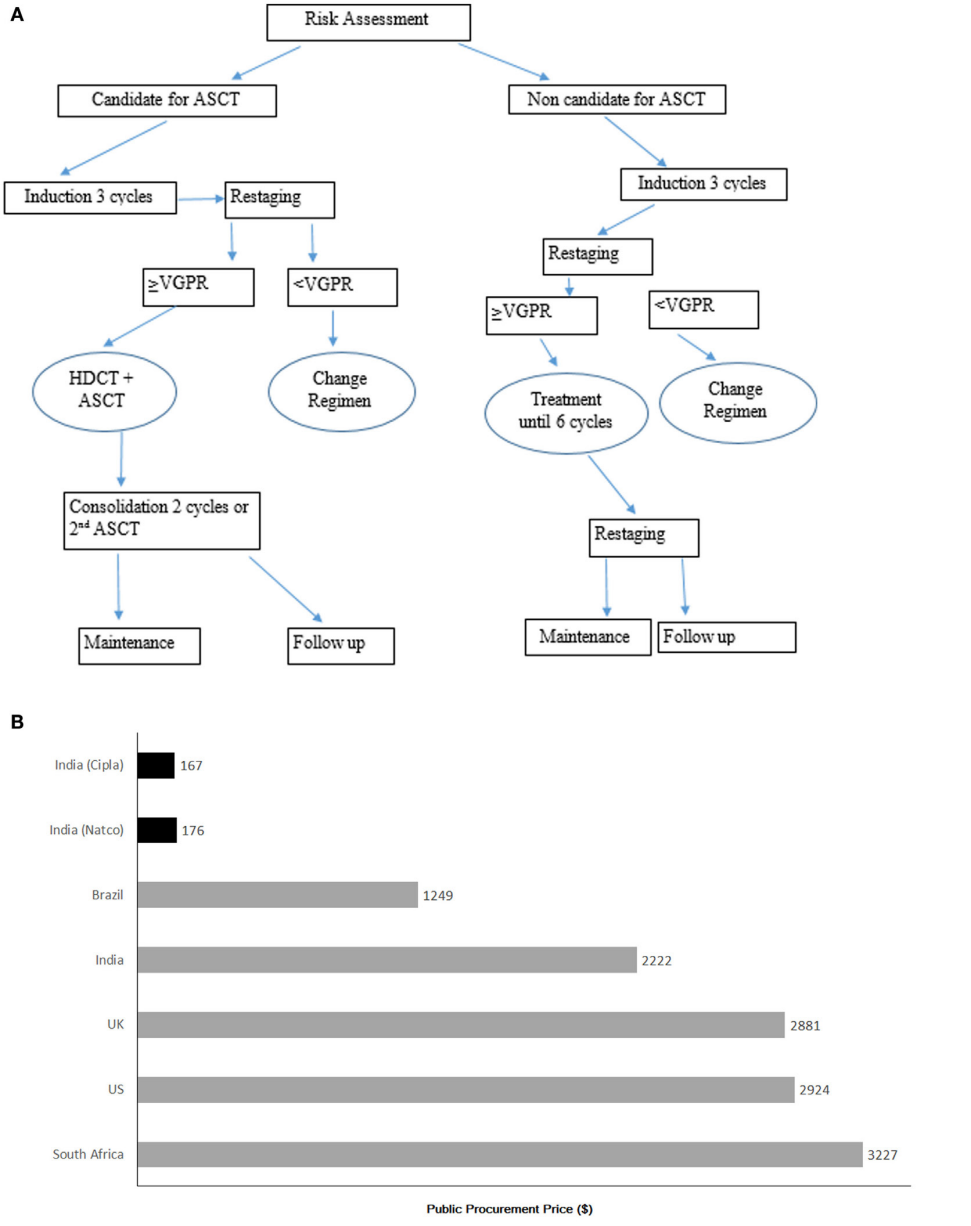

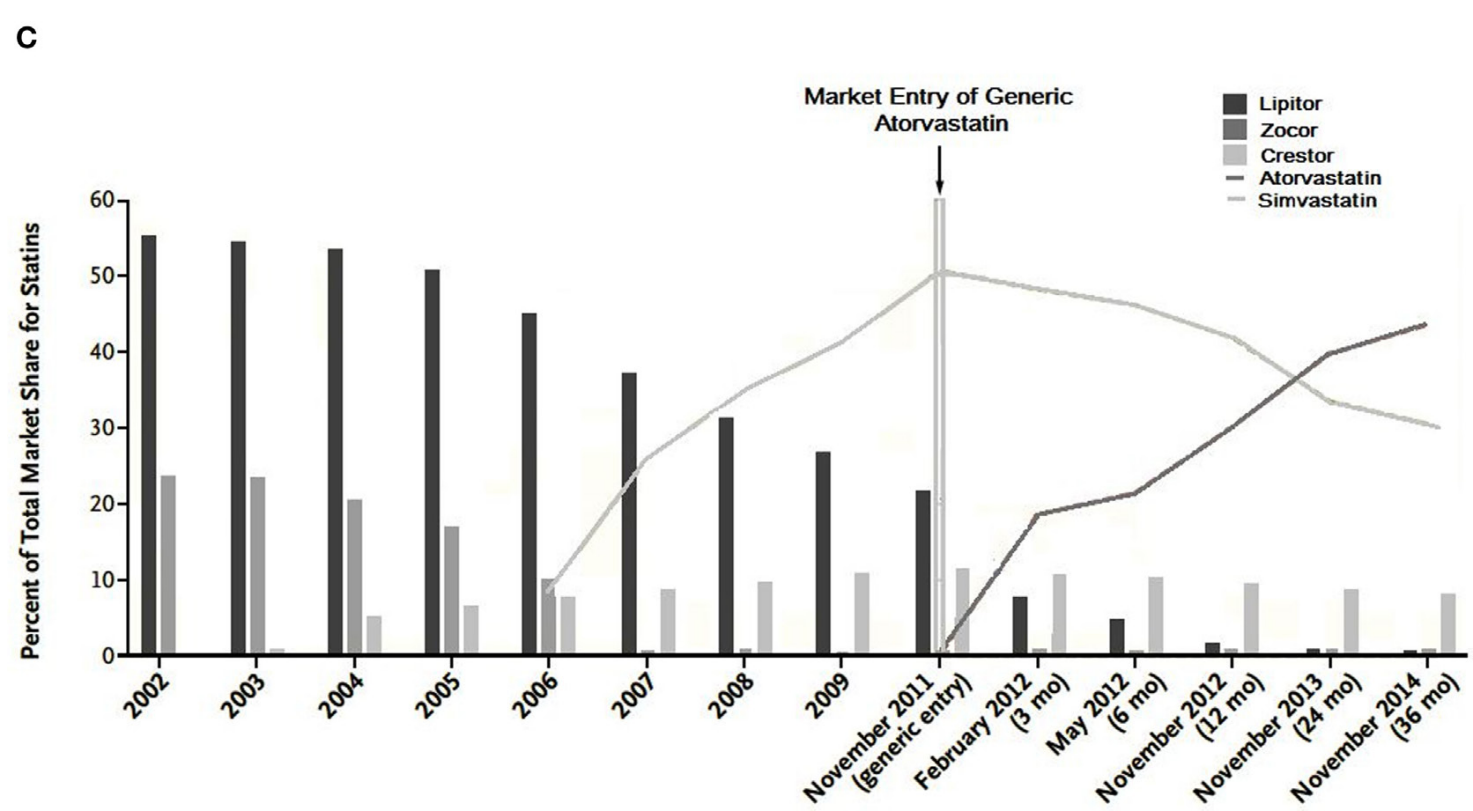
**(A)** Treatment algorithm according to National Guidelines (Ukraine) (10). *ASCT, autologous stan cell transplantation; VGPR, very good partial response; HDCT, high-dose chemotherapy. Currently, routine MM management in Ukraine could vary and differ from guideline due to limitations in health care resources. **(B)** Cost of imatinib brand Gleevac (gray bars) and cost of generic imatinib per patient per month (black bars). Source: MSF-India 2013. **(C)** U.S. Statin market share before and projected market share after the entry of Generic Atorvastatin. Data for 2002 through 2009 are from IMS Health National Prescription Audit.

The treatment strategy is based on NCCN guidelines in India. For the diagnosis and treatment of MM in Ukraine, system of international guidelines [British Committee for Standards in Hematology (BCSH) (8), European Society for Medical Oncology (ESMO) (9), and NCCN guidelines (7)] was incorporated into Ukrainian National Guideline at the end of year 2015 (10). The treatment algorithm for the management of MM according to national requirements is as depicted in the Figure 1A.

Currently routine MM management in Ukraine could vary and differ from guideline due to limitations in health-care resources.

## Management of Multiple Myeloma: Associated Challenges and Limitations

Management of MM in developing economies was another point of interest in the meeting.

### Ukraine (10, 19) and Indian Scenario

#### Access to Healthcare and Optimal Treatment

In *Ukraine*, almost equal proportion of the MM cases are treated at hematology and oncology centers, respectively. Physicians diagnose MM at primary and secondary health-care levels according to National Guideline. MM cases are registered in “National Cancer Registry of Ukraine” or “Indices of Work of Hematological Service in Ukraine.”

In contrast, majority of the health-care system for oncology is private in *India*. Although some of the government set-ups are available, most of the patients have to buy drug on their own.

#### Availability of Diagnostics and Treatment Drugs

##### Diagnostics

As per the order of Ministry of Health of *Ukraine* (10), each patient needs to be diagnosed and treated as per the standard procedure.

For diagnosis, X-ray, computed tomography (CT), and magnetic resonance imaging (MRI) are easily available. However, there are only three centers for positron emission tomography (PET), four centers for serum/urine immuno-fixation and serum/urine protein electrophoresis, three for cytogenetics, and one for free light chain (FLC) assay. All these tests are associated with high costs. Immuno-fixation, bone marrow analysis, and MRI are commonly used modes of diagnosis. PET is not used much often due to high cost factors.

There is only one transplant center in Ukraine for patients who require transplantation. The transplant cost for MM in Ukraine is high, which is unaffordable by patients with lack of state support.

In *India*, most centers have the availability of serum/urine electrophoresis, serum FLC assays, bone marrow, and X-ray survey. Some centers may not have facilities for serum β2-microglobulin. Facilities for fluorescence *in situ* hybridization (FISH) test are not available routinely in most centers (20). Most of the patients do not go for transplant because of its high cost, fear, and social stigma.

A leading Professor of Medical Oncology, Dr. Lalit Kumar, in one of his interview told that in All India Institute of Medical Sciences (AIIMS), Delhi, which is funded by central government, the cost of transplant is less than INR 300,000 (i.e., 6,000 US $). Private hospital charges approximately INR 800,000 (i.e., 16,000 US $) (20). There are 25 regional cancer centers, and more than 15 centers in India which are doing regular stem cell transplants (21).

#### Treatment Options in MM Patients in *Ukraine*

There is not sufficient funding for affording first-line treatment drugs, thus various treatment options, such as thalidomide and bortezomib, are preferred. Bortezomib usage is less due to high cost and lack of funds. Only 20–30% patients are funded by the official program for buying these drugs. Thalidomide is affordable and presently available also as maintenance therapy in Ukraine. Lenalidomide and bortezomib are more commonly used worldwide than thalidomide.

The choice of treatment in different categories of patients is as mentioned below:

*Transplant eligible patients*: vincristine, doxorubicin, and dexamethasone (VAD) are still used in some transplant eligible patients in Ukraine. However, bortezomib/thalidomide-based regimens, such as DVD (pegylated liposomal doxorubicin, bortezomib, and dexamethasone), thalidomide, cyclophosphamide, dexamethasone (TCD), PAD (bortezomib, doxorubicin, dexamethasone), VTD (bortezomib, thalidomide, dexamethasone) etc., are mostly preferred.
*Transplant ineligible patients*: melphalan, prednisolone (MP), high dose of dexamethasone, high-dose MP, VMP (bortezomib, melphalan, prednisolone), VAD, and VTD are preferred.
*In elderly*: low-dose MP, low-dose dexamethasone, TCD, and VCD (bortezomib, cyclophosphamide, dexamethasone) are preferred.
*Relapsed and refractory patients*: the management is challenged by availability of drugs. Mainly three drugs are used upfront (cyclophosphamide, dexamethasone, bortezomib, or thalidomide). In case of patients with lower socio-economic background, best affordable treatment option is provided. Though bortezomib is preferred in triple therapy, the more affordable regimen, such as cyclophosphamide, thalidomide, and dexamethasone (CTD), may be considered. Patients are observed for a long time before starting treatment for relapse and treated only if they become symptomatic. At early relapse or disease progression on therapy, therapy is changed to other, non-cross-resistance, regimens. At late relapse, therapy is chosen taking into account duration of patient response to treatment, toxicities, and received first-line therapy. The following iMiDs are used for relapse treatment: thalidomide, lenalidomide; also bortezomib.

Furthermore, in the meeting, there was a brief discussion on the 10 years of experience of MM treatment with bortezomib at Lviv’s center in Ukraine by Orest Tsyapka, DMS, PhD. He stated that “total 59 and 37 patients were assigned to bortezomib and thalidomide-based regimens, respectively. Fifteen patients received transplant. In 2015, median survival in patients with MM was found to be 74 months. Overall survival (OS) and progression free survival (PFS) were found to be better with bortezomib as compared to thalidomide. Overall survival for naïve patients as well as relapsed/refractory patients treated with bortezomib was again better than thalidomide. Median survival was found to be 59 months in the year 2010 in MM patients, which was found to be increased to 74 months after the use of bortezomib was started.”

#### Treatment Options in MM Patients in *India*

In India, the generics drug market is expanding. Competition among generic companies keeps the price lower for these drugs. There are many Indian companies producing generic drugs in India. However, situation is changing because of patent issues. As a result, very few patients can afford newer molecules such as pomalidomide and carfilzomib.

Majority of patients are treated with a 3-drugs regimen for 6–8 months followed by maintenance. However, once these patients relapse, it is difficult to treat as majority of drugs are already used upfront. Moreover, newer molecules are not available in India.

Majority of patients in India are treated with bortezomib upfront or lenalidomide-based drugs. If a patient has prolonged remission, these drugs can be again used in relapse.

Bortezomib is recommended by NCCN for RRMM: as monotherapy, in combination with PEGylated liposomal doxorubicin, or with panobinostat plus low-dose dexamethasone. The drug results in 55–87% response rates in combination therapies. It does not cause resistance even in case of relapse, and thus retreatment with bortezomib is feasible. A retrospective analysis from a tertiary care center in Hyderabad, India, in newly diagnosed patients suggest that bortezomib is effective and safe as first-line therapy in MM patients. The overall response rate, CR, and VGPR were found to be 88.5, 31.4, and 34.2%, respectively. Bortezomib is effective in reversing renal impairment. The only contraindication is significant neuropathy in upfront use (22).

Lenalidomide is approved for RRMM based on two parallel phase III randomized trials of lenalidomide and dexamethasone vs. dexamethasone alone in patients with RRMM showing significant improvement in median OS. Lenalidomide and dexamethasone are recommended for treatment for RRMM (category 1) in NCCN guidelines. About 65–95% response has been observed with lenalidomide-based regimens.

Thalidomide is not effective at relapse setting. However, it is safe in context of renal failures. It is basically used in combination therapies. Clinical benefit rate of about 70% has been observed with thalidomide in combination regimens with agents, such as vorinostat, bortezomib, or carfilzomib, for refractory MM. Pomalidomide is not available in India but has deeper response and is safer. Similarly, carfilzomib is also not available, which has shown significantly improved median PFS in one of the study.

Other points of discussion were:

- In India, subcutaneous forms are used more, and higher dose is used in subcutaneous than intravenous drugs.
- Combination cytotoxic regimens are reasonable as salvage therapy. Favorable responses have been observed with combination of cyclophosphamide with bortezomib, lenalidomide, or thalidomide.
- Benefit of ASCT in salvage setting is not clear, and thus it should not be considered outside clinical trial setting due to substantial mortality risk. Benefit of ASCT in salvage setting is not clear, and thus it should not be considered outside clinical trial setting due to substantial mortality risk.

## Discussion

As MM has evolved from acute to chronic disease, there is a continuous increase in treatment costs due to longer OS and continual care required during remissions and relapses. Novel agents, such as immune modulators (iMiDs) and PIs, have improved the response rates and survival, but they are given in addition to standard treatments. Additional costs of routine diagnostics, stem cell transplants, and supportive care further pose a tremendous economic burden to the patients and the health-care system. These are important aspects to be considered, especially in emerging economies where there are lack of proper resources and funds (23).

On the basis of most recent published estimates of the average prices in the US, cost of one 21-day cycle of bortezomib was estimated to be $6,450 (3.5 mg vial, quantity 4) or $70,950 per patient (11 cycles) (24). These costs vary as per regimen and further across countries due to variation in pricing agreements and availability of generics (24).

The availability of generic drugs at a comparatively lower cost without compromising quality would be an affordable option.

For example, the steep decline in imatinib costs per patient treatment for chronic myeloid leukemia (CML) per month occurred after its patent expiry and entry of generic version in the market in various countries (Figure 1B) (25). Similarly, the appearance of the generic atorvastatin boosted lipid-lowering treatment economic availability in the US after atorvastatin patent expiry (Figure 1C) (26).

Costs can be reduced further by the use of guideline based therapy, targeted therapies, and therapies that offer lower toxicity, higher survival, and better quality of life. Appropriate supportive care strategies, minimizing hospitalization, and department (ED) visits, decreasing the use of medical care at the end of life are the other ways of cost containment.

Cost containment is an important goal to reduce this burden; however, it should not be the only consideration for treatment choice. Quality of care and an optimal outcome should be the priority for both the managed care organizations and patients.

A few pharmacoeconomic models and studies have been done to study the cost-effectiveness.

Garrison et al. conducted a pharmacoeconomic analysis for transplant ineligible patients comparing MP with novel regimens, such as VMP, melphalan/prednisone/thalidomide (MPT), melphalan/prednisone/lenalidomide (MPR), and MPR-R. The study concluded that VMP is likely to be more cost-effective and cost saving as compared to other regimens (27).

In case of RRMM, bortezomib is comparatively a more cost-effective treatment (in terms of cost/life year gained) as compared to other available options. It provides an additional life year at a cost of $45,356. Also its incremental cost-effectiveness ratio (ICER) remains favorable (28).

In two studies done by the investigators in Ukraine, bortezomib was found to be the most economically significant treatment. A retrospecive analysis of the pilot study was performed in Ukraine at Kiev center. The average annual total cost of pharmacotherapy in MM patients in 2012 was equivalent to 2,297,984 EUR of which bortezomib had taken the major part of total costs (29, 30).

Evaluations of newer, advanced therapies are lacking despite their significant contribution in improving quality of life and survival of patients and therefore warranted in future.

With the rapidly evolving treatment landscape and forthcoming options and guidelines, a lot of flexibility is allowed in prescribing, as described under NCCN guidelines. Formulating clinical pathways using an evidence-based approach in line with guidelines might be a good option. This might also reduce local, regional, and national variations in practice.

A tremendous effort is required to improve the health-care access to the MM patients. Quality cancer care systems should be implemented with more defined networking amongst the health-care providers, medical oncologists, hematologists, surgeons, etc. and strong decision-support strategies. More transplant or transfusion centers need to be established, and a larger number of oncologists and surgeons are required to be added to the current pool.

Furthermore, an improved collaboration between all the stakeholders, health-care professionals, pharmaceutical industry, regulatory agencies, and managed care organizations is the need of hour to fill the current gaps in the management of MM in emerging or developing nations.

## Conclusion

Cost reduction, improved healthcare access, enhanced collaboration, and common treatment strategies are few issues, which need immediate attention in the management of MM.

The recent advancements in the management of MM and possible addition of second- or third-generation PIs and iMiDs in the near future will increase the economic burden. Whether this increased burden is justified by the health gain produced is the question, which needs further investigation through more pharmacoeconomic studies and head-to-head trials between various agents for determining their cost-effectiveness and overall benefit.

## Author Contributions

India and Ukraine Haemato-oncology Group (DC, DB, IK, IKK, OT, RN, and VK) – equal contribution by all authors, listed in alphabetical order. AG developed the structure and arguments for the paper; SA contributed in providing graphs; NG drafted the manuscript; all the authors made critical revisions and approved the manuscript.

## India and Ukraine Haemato-Oncology Group

Dharma Choudhary, BLK Super Specialty Hospital, New Delhi, India, dharmabmt@yahoo.in. Dinesh Bhurani, Heamato-Oncology Services & Bone Marrow Transplant Unit, Rajiv Gandhi Cancer Institute & Research Centre, Delhi, India, bhurani@gmail.com. Iryna Kryachok, National Cancer Institute, Kiev, Ukraine, irina.kryachok@gmail.com. Iryna Koren’kova, Kiev Center for Bone Marrow Transplantation, Kiev, Ukraine, irina_br@mail.ru. Orest Tsyapka, Institute of Blood Pathology and Transfusion Medicine, Lviv, Ukraine, tsyapka@yahoo.com. Rahul Naithani, Hematology & Bone Marrow Transplantation, Max Hospitals, New Delhi, India, dr_rahul6@hotmail.com. Viktor Kozlov, Odessa Regional Clinical Hospital, Odessa, Ukraine, v_kozlov@ukr.net.

## Conflict of Interest Statement

AG, MM, YB, NG, and SA are employees of Dr. Reddy’s Laboratories Ltd., India. The reviewer SG and handling Editor declared their shared affiliation, and the handling Editor states that the process nevertheless met the standards of a fair and objective review.
